# Diagnostic and prognostic value of CEA, CA19–9, AFP and CA125 for early gastric cancer

**DOI:** 10.1186/s12885-017-3738-y

**Published:** 2017-11-09

**Authors:** Fan Feng, Yangzi Tian, Guanghui Xu, Zhen Liu, Shushang Liu, Gaozan Zheng, Man Guo, Xiao Lian, Daiming Fan, Hongwei Zhang

**Affiliations:** 1 Division of Digestive Surgery, Xijing Hospital of Digestive Diseases, the Fourth Military Medical University, 127 West Changle Road, 710032, , Xian, Shaanxi China; 2 Department of Dermatology, Xijing Hospital, the Fourth Military Medical University, 127 West Changle Road, 710032, , Xian, Shaanxi China

**Keywords:** Early gastric cancer, Diagnosis, Prognosis, Tumor marker

## Abstract

**Background:**

The diagnostic and prognostic significance of carcinoembryonic antigen (CEA), carbohydrate associated antigen 19–9 (CA19–9), alpha-fetoprotein (AFP) and cancer antigen 125 (CA125) in early gastric cancer have not been investigated yet. Thus, the present study aimed to explore the diagnostic and prognostic significance of the four tumor markers for early gastric cancer.

**Methods:**

From September 2008 to March 2015, 587 early gastric cancer patients were given radical gastrectomy in our center. The clinicopathological characteristics were recorded. The association between levels of CEA and CA19–9 and clinicopathological characteristics and prognosis of patients were analyzed.

**Results:**

There were 444 men (75.6%) and 143 women (24.4%). The median age was 57 years (ranged 21–85). The 1-, 3- and 5-year overall survival rate was 99.1%, 96.8% and 93.1%, respectively. The positive rate of CEA, CA19–9, AFP and CA125 was 4.3%, 4.8%, 1.5% and 1.9%, respectively. The positive rate of all markers combined was 10.4%. The associations between the clinicopathological features and levels of CEA and CA19–9 were analyzed. No significant association was found between CEA level and clinicopathological features. However, elevated CA19–9 level was correlated with female gender and presence of lymph node metastasis. Age > 60 years old, presence of lymph node metastasis and elevation of CEA level were independent risk factors for poor prognosis of early gastric cancer.

**Conclusions:**

The positive rates of CEA, CA19–9, APF and CA125 were relatively low for early gastric cancer. Elevation of CA19–9 level was associated with female gender and presence of lymph node metastasis. Elevation of CEA level was an independent risk factor for the poor prognosis of early gastric cancer.

## Background

Gastric cancer is the fourth commonest malignancy and the second leading cause of tumor related death all over the world [1]. Early gastric cancer is a lesion only invading mucosa or submucosa, with or without lymph node metastasis (LNM) [2]. Early diagnosis of gastric cancer is critical for optimal treatment. The ratio of early gastric cancer at diagnosis is increasing with advanced techniques and screening programs [3]. As detection of serum tumor markers are more convenient than other approaches, they are widely applied in early diagnosis of gastric cancer [4]. Unfortunately, the optimal serum biomarker for the detection of early gastric cancer is still under investigation [5].

The prognosis of early gastric cancer is favorable after radical gastrectomy, with a 5-year overall survival rate exceed 97% [6]. A variety of factors have been recognized as prognostic factors for early gastric cancer, including tumor size, differentiation status, tumor depth, LNM and vessel involvement [7]. In addition, tumor markers including CEA [8], CA19–9 [9], and AFP [10] were demonstrated to be prognostic factors for gastric cancer. However, prognostic significance of these markers for early gastric cancer have not been investigated yet.

Given this situation, the present study aims to explore the diagnostic and prognostic significance of CEA, CA19–9, AFP and CA125 for early gastric cancer.

## Methods

This study was carried out in the Xijing Hospital of Digestive Diseases, the Fourth Military Medical University. From September 2008 to March 2015, 587 early gastric cancer patients with radical gastrectomy were enrolled in our present study. This study was approved by the Ethics Committee of Xijing Hospital, and written informed consent was obtained from all patients before surgery.

All patients were treated with proximal, distal or total D2 gastrectomy. The procedure was based on the Japanese Gastric Cancer Treatment Guidelines [11]. Tumor depth and LNM were defined by pathologists in the department of pathology according to the TNM classification.

Preoperative data including gender, age, tumor location, serum CEA, CA19–9, AFP and CA125 levels were recorded. Tumor size, differentiation status, tumor depth and LNM were collected based on pathology reports. Patients were followed up till November 2016 every 3 months.

The tumor markers were detected within 7 days before surgery. The cut off value of CEA, CA19–9, AFP and CA125 levels were 5 ng/ml, 27 U/ml, 8.1 ng/ml, 35 U/ml. The positive rates of tumor markers were defined as number of cases with elevated markers divided by total number of cases. The positive rates of combined markers were defined as number of cases with elevation in any of the markers divided by total number of cases.

Data were analyzed using SPSS 22.0 for Windows (SPSS Inc., Chicago, IL, USA). Discrete variables were analyzed by Fisher’s exact test or Chi-square test. Significant prognostic factors for early gastric cancer patients identified by univariate analysis were further assessed with multivariate analysis using the Cox’s proportional hazards regression model. Survival curves for overall survival were obtained using the Kaplan-Meier method**.** The *P* value less than 0.05 was considered to be statistically significant.

## Results

**Fig. 1:**
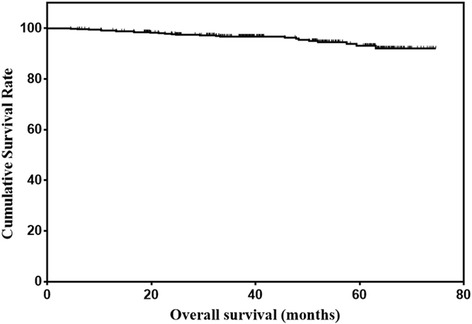
Overall survival of early gastric cancer patients

The features of the entire cohort were summarized in Table 1. There were 444 men (75.6%) and 143 women (24.4%). The median age was 57 years (21–85 years). The median follow up time was 39 months (5–75 months). The total number of death during follow up was 25. The 1-, 3- and 5-year overall survival rate was 99.1%, 96.8% and 93.1%, respectively (Fig. 1).

**Table 1:** Clinicopathological characteristics of early gastric cancer patients

Characteristics	No. of patients	Percent
Gender		
Male	444	75.6
Female	143	24.4
Age		
≤ 60	368	62.7
> 60	219	37.3
Tumor location		
Upper third	102	17.4
Middle third	100	17.0
Lower third	385	65.6
Tumor size (cm)		
≤ 2	365	62.2
> 2	222	37.8
Pathological type		
Well differentiated	186	31.7
Moderately differentiated	163	27.8
Poorly differentiated	220	37.5
Signet ring cell or Mucinous	18	3.0
Tumor depth		
T1a	255	43.4
T1b	332	56.6
Lymph node metastasis		
N0	495	84.3
N1	55	9.4
N2	29	4.9
N3	8	1.4

The positive rates of the four markers were summarized in Table 2. The positive rate of CEA, CA19–9, AFP and CA125 level were 4.3%, 4.8%, 1.5% and 1.9%, respectively. The highest positive rate was 8.2% for combination of two markers (CA19–9 and CEA), 9.4% for combination of three markers (CA19–9, CEA and AFP or CA19–9, CEA and CA125), and 10.4% for combination of all four markers.

**Table 2:** Positive rates of single and combined tumor markers in early gastric cancer patients

Tumor marker		CA19–9	AFP	CA125
CEA	25(4.3%)	48(8.2%)	31(5.3%)	35(6.0%)
CA19–9	28(4.8%)		37(6.3%)	33(5.6%)
AFP	9(1.5%)			20(3.4%)
CA125	11(1.9%)			
CEA + CA19–9			55(9.4%)	55(9.4%)
CEA + AFP				41(7.0%)
CA19–9 + AFP				44(7.5%)
CEA + CA19–9 + AFP				61(10.4%)

Considering the extremely low positive rates of AFP and CA125, we only analyzed the correlation between level of CEA and CA19–9 and clinicopathological features. No association was found between CEA level and clinicopathological features (Table 3). However, elevation of CA19–9 level was correlated with female gender and presence of LNM (Table 4).

**Table 3:** Comparison of clinicopathological characteristics between two groups stratified by CEA level

Characteristics	CEA(−)	CEA(+)	P
Gender			
Male	422	22	0.161
Female	140	3	
Age			
≤ 60	351	17	0.675
> 60	211	8	
Tumor location			
Upper third	99	3	0.744
Middle third	95	5	
Lower third	368	17	
Tumor size (cm)			
≤ 2	346	19	0.205
> 2	216	6	
Pathological type			
Well differentiated	180	6	0.537
Moderately differentiated	153	10	
Poorly differentiated	212	8	
Signet ring cell or Mucinous	17	1	
Tumor depth			
T1a	245	9	0.539
T1b	317	16	
Lymph node metastasis			
N0	474	21	0.698
N1	53	2	
N2	28	1	
N3	7	1

**Table 4:** Comparison of clinicopathological characteristics between two groups stratified by CA 19–9 level

Characteristics	CA19–9(−)	CA19–9(+)	P
Gender			
Male	428	16	0.025
Female	131	12	
Age			
≤ 60	351	17	0.843
> 60	208	11	
Tumor location			
Upper third	95	7	0.543
Middle third	96	4	
Lower third	368	17	
Tumor size (cm)			
≤ 2	345	20	0.327
> 2	214	8	
Pathological type			
Well differentiated	178	8	0.936
Moderately differentiated	156	7	
Poorly differentiated	208	12	
Signet ring cell or Mucinous	17	1	
Tumor depth			
T1a	243	12	1.000
T1b	316	16	
Lymph node metastasis			
N0	475	20	0.020
N1	52	3	
N2	26	3	
N3	6	2	

**Fig. 2:**
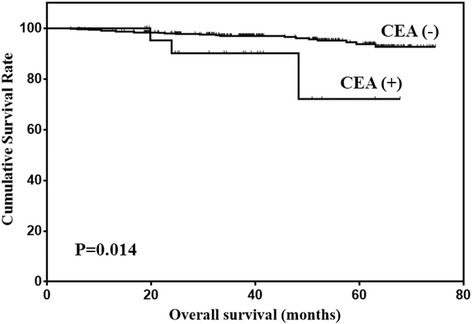
Overall survival of early gastric cancer patients stratified by CEA level

**Fig. 3:**
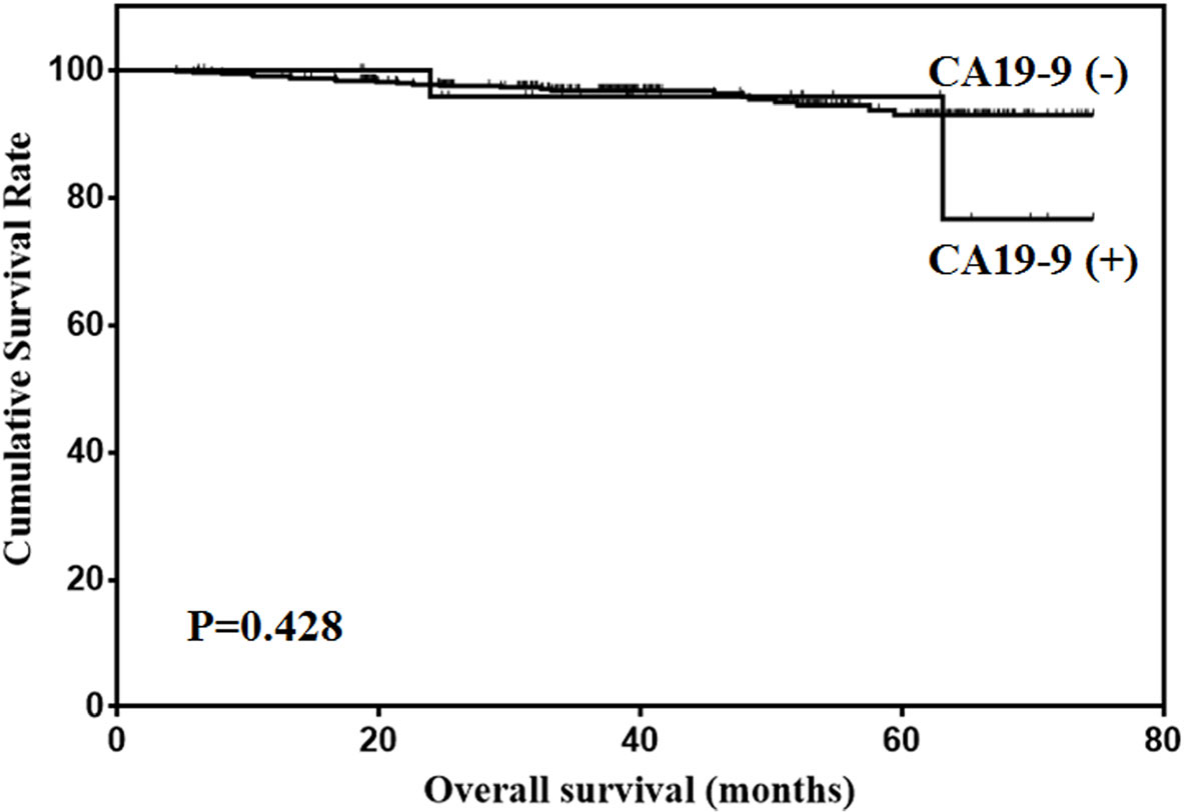
Overall survival of early gastric cancer patients stratified by CA19–9 level

Prognostic factors for early gastric cancer patients were analyzed using univariate analysis (Table 5). The results showed that age, LNM and CEA level were prognostic factors for early gastric cancer. The variables used for adjustment in the multivariate analyses were age, LNM and CEA level. The results showed that age, LNM and CEA level were independent prognostic factors according to multivariate analysis (Table 6). The overall survival of early gastric cancer patients according to the levels of CEA and CA19–9 were shown in Figs. 2 and 3.

**Table 5:** Univariate analysis of prognostic factors for early gastric cancer

Prognostic factors	β	Hazard ratio (95% CI)	*P* value
Gender	0.105	1.110(0.443–2.783)	0.824
Age	1.195	3.304(1.425–7.661)	0.005
Tumor location	−0.283	0.754(0.478–1.189)	0.224
Tumor size	−0.687	0.503(0.201–1.260)	0.142
Pathological type	−0.388	0.679(0.431–1.067)	0.093
Tumor depth	0.736	2.088(0.831–5.241)	0.117
Lymph node metastasis	0.577	1.781(1.124–2.821)	0.014
CEA	1.404	4.070(1.208–13.713)	0.024
CA19–9	0.576	1.779(0.419–7.546)	0.435
AFP	−3.019	0.049(0.000–590,647.114)	0.717
CA125	0.740	2.095(0.283–15.490)	0.469

**Table 6:** Multivariate analysis of prognostic factors for early gastric cancer

Prognostic factors	β	Hazard ratio (95% CI)	*P* value
Age	1.379	3.971(1.671–9.435)	0.002
Lymph node metastasis	0.682	1.978(1.248–3.136)	0.004
CEA	1.284	3.611(1.065–12.245)	0.039

## Discussion

Serum tumor markers are widely applied in the diagnosis, treatment effect assessment and disease monitoring [12]. Up to date, a series of studies have explored the diagnostic and prognostic value of various serum tumor markers for gastric cancer [5]. However, no study has explored the diagnostic and prognostic value of serum tumor markers for early gastric cancer. Our present study found that the positive rates of serum CEA, CA19–9, APF and CA125 were relatively low for early gastric cancer. Elevation of CA19–9 level was correlated with female gender and presence of LNM. Elevation of CEA level was an independent risk factor for the poor prognosis of early gastric cancer.

The positive rates of the four markers for early gastric cancer varied widely. It was reported that the positive rate was 4.4%–15.4% for CEA [13–15], 11.7% for CA19–9 [15], 2.5%–3.3% for AFP [16, 17] and 6.7% for CA125 [17]. In the present study, the positive rates of all four tumor markers were lower than previous reports. Even with the combination of four tumor markers, the positive rate was only 10.4%. This indicated that the diagnostic value of the four tumor markers was extremely low for early gastric cancer.

A strong correlation between elevated tumor markers and clinicopathological features has been reported previously. It was reported that serum CEA level was correlated with tumor depth, LNM [13] and liver metastasis [18]. Other studies have reported that CA19–9 level was correlated with tumor depth, LNM and tumor stage [19, 20]. However, the association between tumor markers and the clinicopathological features of early gastric cancer has not been investigated yet. In our present study, no association was found between CEA level and clinicopathological features. However, elevation of CA19–9 level was correlated with female gender and presence of LNM.

Early gastric cancer has a favorable outcome after radical gastrectomy. The preoperative tumor markers have been reported as valuable predictors for the prognosis of gastric cancer. A meta-analysis containing 14,651 gastric cancer patients demonstrated that serum CEA level was an independent prognostic factor for gastric cancer [8]. Another meta-analysis revealed that CEA protein and mRNA levels in peritoneal lavage were associated with peritoneal recurrence after radical gastrectomy [21]. A meta-analysis containing 11,408 gastric cancer patients showed that elevated serum CA19–9 level was correlated with poor prognosis [22]. Elevated AFP level was reported to be associated with liver metastasis and poor prognosis of gastric cancer [10, 23, 24]. Elevation of peritoneal lavage CA125 level was correlated with peritoneal dissemination and poor outcomes of gastric cancer [25]. However, the prognostic value of these tumor markers for early gastric cancer was unclear. In our study, considering the extremely low positive rate of AFP and CA125 level, only the prognostic significance of CEA and CA19–9 level were analyzed. The results showed that serum CEA level was an independent prognostic factor for early gastric cancer. However, serum CA19–9 level had no prognostic significance.

There are some limitations in our study. Firstly, we did not evaluate the predictive value of postoperative levels of serum tumor markers for recurrence patterns and prognosis of early gastric cancer. Secondly, the sample size was not large enough, and the positive rate of tumor markers was relatively low, which may result in bias during analysis. Thirdly, mortality was extremely low in early gastric cancer, which will influence the prognostic significance analysis of tumor markers.

## Conclusions

The positive rates of CEA, CA19–9, APF and CA125 were relatively low for early gastric cancer. Elevation of CA19–9 level was associated with female gender and presence of lymph node metastasis. Elevation of CEA level was an independent risk factor for the poor prognosis of early gastric cancer.

## Abbreviations

AFP: alpha-fetoprotein
CA125: cancer antigen 125
CA19–9: carbohydrate associated antigen 19–9
CEA: carcinoembryonic antigen
LNM: lymph node metastasis

## References

[1] Jemal A, Bray F, Center MM, Ferlay J, Ward E, Forman D (2011). Global cancer statistics. CA Cancer J Clin.

[2] Feng F, Sun L, Xu G, Cai L, Hong L, Yang J (2015). Is it reasonable to treat early gastric cancer with mucosal infiltration and well differentiation by endoscopic submucosal resection?. J Gastrointest Surg.

[3] Zhu L, Qin J, Wang J, Guo T, Wang Z, Yang J (2016). Early gastric cancer. Current Advances of Endoscopic Diagnosis and Treatment Gastroenterol Res Pract.

[4] Tian SB, JC Y, Kang WM, Ma ZQ, Ye X, Cao ZJ (2014). Combined detection of CEA, CA 19-9, CA 242 and CA 50 in the diagnosis and prognosis of resectable gastric cancer. Asian Pac J Cancer Prev.

[5] Jin Z, Jiang W, Wang L (2015). Biomarkers for gastric cancer. Progression in early diagnosis and prognosis (review). Oncol Lett.

[6] Pyo JH, Lee H, Min BH, Lee JH, Choi MG, Lee JH (2016). Long-term outcome of endoscopic resection vs. surgery for early gastric cancer: a non-inferiority-matched cohort study. Am J Gastroenterol.

[7] Huang B, Wang Z, Xing C, Sun Z, Zhao B, Long-term XH (2011). Survival results and prognostic factors of early gastric cancer. EXP THER MED.

[8] Deng K, Yang L, Hu B, Wu H, Zhu H, Tang C (2015). The prognostic significance of pretreatment serum CEA levels in gastric cancer: a meta-analysis including 14651 patients. PLoS One.

[9] Xiao J, He X, Wang Z, Hu J, Sun F, Qi F (2014). Serum carbohydrate antigen 19-9 and prognosis of patients with gastric cancer. Tumour Biol.

[10] Liu X, Cheng Y, Sheng W, Lu H, Xu Y, Long Z (2010). Clinicopathologic features and prognostic factors in alpha-fetoprotein-producing gastric cancers: analysis of 104 cases. J Surg Oncol.

[11] Japanese gastric cancer treatment guidelines 2010 (ver. 3) (2011). Gastric Cancer.

[12] Rodriguez-Enriquez S, Pacheco-Velazquez SC, Gallardo-Perez JC, Marin-Hernandez A, Aguilar-Ponce JL, Ruiz-Garcia E (2011). Multi-biomarker pattern for tumor identification and prognosis. J Cell Biochem.

[13] Park SH, Ku KB, Chung HY, Yu W (2008). Prognostic significance of serum and tissue carcinoembryonic antigen in patients with gastric adenocarcinomas. Cancer Res Treat.

[14] Wang W, Chen XL, Zhao SY, YH X, Zhang WH, Liu K (2016). Prognostic significance of preoperative serum CA125, CA19-9 and CEA in gastric carcinoma. Oncotarget.

[15] Liang Y, Wang W, Fang C, Raj SS, Hu WM, Li QW (2016). Clinical significance and diagnostic value of serum CEA, CA19-9 and CA72-4 in patients with gastric cancer. Oncotarget.

[16] Wang D, Li C, Xu Y, Xing Y, Qu L, Guo Y (2015). Clinicopathological characteristics and prognosis of alpha-fetoprotein positive gastric cancer in Chinese patients. Int J Clin Exp Pathol.

[17] He CZ, Zhang KH, Li Q, Liu XH, Hong Y, Lv NH (2013). Combined use of AFP, CEA, CA125 and CAl9-9 improves the sensitivity for the diagnosis of gastric cancer. BMC Gastroenterol.

[18] Ucar E, Semerci E, Ustun H, Yetim T, Huzmeli C, Gullu M (2008). Prognostic value of preoperative CEA, CA 19-9, CA 72-4, and AFP levels in gastric cancer. Adv Ther.

[19] Sisik A, Kaya M, Bas G, Basak F, Alimoglu OCEA (2013). CA 19-9 are still valuable markers for the prognosis of colorectal and gastric cancer patients. Asian Pac J Cancer Prev.

[20] Kochi M, Fujii M, Kanamori N, Kaiga T, Kawakami T, Aizaki K (2000). Evaluation of serum CEA and CA19-9 levels as prognostic factors in patients with gastric cancer. Gastric Cancer.

[21] Xiao Y, Zhang J, He X, Ji J, Wang G (2014). Diagnostic values of carcinoembryonic antigen in predicting peritoneal recurrence after curative resection of gastric cancer: a meta-analysis. Ir J Med Sci.

[22] Song YX, Huang XZ, Gao P, Sun JX, Chen XW, Yang YC, et al. Clinicopathologic and prognostic value of serum carbohydrate antigen 19-9 in gastric cancer: a meta-analysis. Dis Markers 2015, 2015:549843. 10.1155/2015/549843 PMC4631884 26576068

[23] Chen Y, Qu H, Jian M, Sun G, He Q (2015). High level of serum AFP is an independent negative prognostic factor in gastric cancer. Int J Biol Markers.

[24] Zuo C, An JQ (2015). Analysis on clinical characteristics and prognosis of patients with serum alpha-fetoprotein-positive gastric cancer. Minerva Med.

[25] Yamamoto M, Baba H, Toh Y, Okamura T, Maehara Y (2007). Peritoneal lavage CEA/CA125 is a prognostic factor for gastric cancer patients. J Cancer Res Clin Oncol.

